# Personalizing Polymyxin B Dosing Using an Adaptive Feedback Control Algorithm

**DOI:** 10.1128/AAC.00483-18

**Published:** 2018-06-26

**Authors:** Elizabeth A. Lakota, Cornelia B. Landersdorfer, Roger L. Nation, Jian Li, Keith S. Kaye, Gauri G. Rao, Alan Forrest

**Affiliations:** a University at Buffalo, Buffalo, New York, USA; b Institute for Clinical Pharmacodynamics, Schenectady, New York, USA; c Drug Delivery, Disposition and Dynamics, Monash Institute of Pharmaceutical Sciences, Monash University, Parkville Victoria, Australia; d Centre for Medicine Use and Safety, Faculty of Pharmacy and Pharmaceutical Sciences, Monash University, Parkville, Victoria, Australia; e Monash Biomedicine Discovery Institute, Department of Microbiology, Monash University, Victoria, Australia; f Department of Internal Medicine, Division of Infectious Diseases, University of Michigan Medical School, Ann Arbor, Michigan; g Division of Pharmacotherapy and Experimental Therapeutics, Eshelman School of Pharmacy, University of North Carolina at Chapel Hill, Chapel Hill, North Carolina

**Keywords:** polymyxins, nephrotoxicity, adaptive feedback control, multidrug-resistant organisms

## Abstract

Polymyxin B is used as an antibiotic of last resort for patients with multidrug-resistant Gram-negative bacterial infections; however, it carries a significant risk of nephrotoxicity. Herein we present a polymyxin B therapeutic window based on target area under the concentration-time curve (AUC) values and an adaptive feedback control algorithm (algorithm) which allows for the personalization of polymyxin B dosing. The upper bound of this therapeutic window was determined through a pharmacometric meta-analysis of polymyxin B nephrotoxicity data, and the lower bound was derived from murine thigh infection pharmacokinetic (PK)/pharmacodynamic (PD) studies. A previously developed polymyxin B population pharmacokinetic model was used as the backbone for the algorithm. Monte Carlo simulations (MCS) were performed to evaluate the performance of the algorithm using different sparse PK sampling strategies. The results of the nephrotoxicity meta-analysis showed that nephrotoxicity rate was significantly correlated with polymyxin B exposure. Based on this analysis and previously reported murine PK/PD studies, the target AUC_0–24_ (AUC from 0 to 24 h) window was determined to be 50 to 100 mg · h/liter. MCS showed that with standard polymyxin B dosing without adaptive feedback control, only 71% of simulated subjects achieved AUC values within this window. Using a single PK sample collected at 24 h and the algorithm, personalized dosing regimens could be computed, which resulted in >95% of simulated subjects achieving AUC_0–24_ values within the target window. Target attainment further increased when more samples were used. Our algorithm increases the probability of target attainment by using as few as one pharmacokinetic sample and enables precise, personalized dosing in a vulnerable patient population.

## INTRODUCTION

Polymyxins were developed in the 1950s to treat Gram-negative bacterial infections. Although efficacious, they were soon found to have a narrow therapeutic window and cause significant nephrotoxicity (1, 2). Consequently, their use declined when antibiotics perceived at the time to be less toxic, such as the aminoglycosides, became available. Today, there are growing numbers of infections with difficult-to-treat multidrug-resistant (MDR) bacteria. As a result, polymyxins are again being used but as drugs of last resort to treat Gram-negative MDR infections, oftentimes in critically ill patients.

Due to their toxicity and use in a vulnerable patient population (very ill patients at an increased risk for infections due to MDR pathogens), polymyxins must be optimally dosed to achieve exposures that minimize nephrotoxicity while maximizing bacterial eradication. Even a modest degree of pharmacokinetic (PK) variability renders the selection of a standard dosing regimen for drugs with narrow therapeutic windows, such as the polymyxins, difficult. To address this problem with other drugs, adaptive feedback control algorithms (3–5) have been proposed. Adaptive feedback control strategies attempt to derive patient-specific PK information using sparse PK samples and population PK models in order to optimize dosing and clinical outcome. A priori PK parameter estimates, resulting from an established population PK model, are used to develop a maximum a posteriori probability (MAP) Bayesian PK parameter estimator. The MAP Bayesian estimator and PK samples from the subject of interest are then used to estimate the subject's individual PK parameters. Based on the subject-specific PK parameter estimates, new personalized doses can be computed to optimize drug exposure.

Two polymyxins are currently available for clinical use, polymyxin B and colistin (polymyxin E), which are almost identical apart from a single amino acid difference (6). In contrast to colistin, which is administered as an inactive prodrug (colistimethate), polymyxin B is delivered as an active compound and requires no bioactivation, which leads to less complex and less variable PK. In addition, PK sample collection and processing are much simpler for polymyxin B than for colistin (7). Further, recent clinical studies have suggested that polymyxin B is less nephrotoxic than colistin (8–12), indicating a potentially wider therapeutic window. For these reasons, polymyxin B is better suited for optimization based on an adaptive feedback control algorithm.

Herein we describe a method to individually optimize polymyxin B doses. Our first objective was to develop a target area under the concentration-time curve (AUC) window for polymyxin B given that the AUC/MIC ratio is the pharmacokinetic/pharmacodynamic index most associated with polymyxin efficacy (13–15) and AUC is associated with nephrotoxicity (16–18). Our second objective was to develop a novel adaptive feedback control algorithm to allow for personalized polymyxin B dosing and to test the algorithm *in silico*.

## RESULTS

### Pharmacometric meta-analysis of nephrotoxicity.

Nineteen primary research articles contained data on nephrotoxicity rates associated with administration of intravenous polymyxin B. Two articles did not provide sufficient information regarding the polymyxin B doses received by patients and were excluded from this analysis. A summary of the 17 included articles is provided in Table 1, and a summary of the polymyxin B Monte Carlo simulations based on these studies is shown in Table 2. The median (range) simulated 75th percentile of steady-state 24-h AUC (ssAUC_0–24_) values across studies was 80.4 mg · h/liter (58.9 to 117 mg · h/liter).

**TABLE 1:** Summary of polymyxin B-associated nephrotoxicity literature included in the pharmacometric nephrotoxicity meta-analysis^*a*^

Author (yr) (reference)	No. of subjects receiving PMB^*b*^	Institution PMB dosing recommendations (mg/kg/day)	Daily PMB dose (mg/day)	Wt (kg)	Nephrotoxicity definition	% nephrotoxicity incidence (no. of patients affected/total no.)
Ouderkirk et al. (2003) (32)	50	1.5–2.5	Mean, 110	NA	2-fold ↑ in SCr to ≥2 mg/dl	14 (7/50)
Holloway et al. (2006) (33)	31	NA	Median, 130	NA	1.5-fold ↑ in SCr, ↑ in SCr of ≥0.5 mg/dl, or 50% reduction in CL_CR_	22.5 (7/31)
Teng et al. (2007) (21)	27	NA	Mean, 62.9	NA	1.5-fold ↑ in SCr, ↑ in SCr of ≥0.5 mg/dl, or 50% reduction in CL_CR_	18.5 (5/27)
Pastewski et al. (2008) (20)	11	1.5–2, 1.25 if CL_CR_ is 30–80 ml/min, 0.5 if CL_CR_ is <30 ml/min	Mean, 84	NA	↑ in SCr of ≥0.5 mg/dl or 50% reduction in CL_CR_	54.5 (6/11)
Ramasubban et al. (2008) (34)	45	1.5–2	Mean, 120	NA	↑ in SCr by 0.5 mg/dl	8.89 (4/45)
Mendes et al. (2009) (35)	114	NA	Mean, 96.7	NA	If baseline SCr is <1.5 mg/dl, SCr ↑ to ≥1.8; if baseline SCr is >1.5 and <4, 1.5-fold ↑in SCr	21.9 (25/114)
Oliveira et al. (2009) (36)	30	NA	Median, 100	NA	2-fold ↑ in SCr or ↑ in SCr of ≥1 mg/dl if initial SCr is >1.4 mg/dl	27 (8/30)
Elias et al. (2010) (19)	235	NA	Median, 150	NA	Mild, 50–100% ↑ in SCr; moderate, ≥100% ↑ in SCr but no dialysis; severe, need for dialysis	Mild, 13.6; moderate, 26.4; severe, 21.9
Kvitko et al. (2011) (37)	45	NA	Mean, 141	NA	Stage 1, 1.5- to 2-fold ↑ in SCr; stage 2, ≥2-fold ↑ in SCr	Stage 1, 11 (4/45); stage 2, 24 (11/45)
Esaian et al. (2012) (38)	115	1.5–2.5, adjust for renal dysfunction	Median, 100	Median, 69	Meeting any of the RIFLE criteria	Risk, 48 (55/115), injury, 31 (36/115); failure, 17 (19/115)
Kubin et al. (2012) (39)	73	2.5–3, 1–1.5 if CL_CR_ is <80 ml/min	Median, 180	Median, 76.4	Meeting any of the RIFLE criteria	Risk, 27.4 (20/73); injury or failure, 20 (24/73)
Tuon et al. (2013) (9)	96	NA	Median, 200	NA	Stage 1, 1.5- to 2-fold ↑ in SCr or SCr ↑ of 0.3 mg/dl; stage 2, 2- to 3-fold ↑ in SCr; stage 3, >3-fold ↑in SCr or SCr of ≥4 mg/dl with acute rise of ≥0.5 mg/dl	Stage 1, 11.5 (11/96); stage 2, 8.33 (8/96); stage 3, 1.04 (1/96)
Nandha et al. (2013) (40)	32	1.5–2.5	Mean, 111	NA	Meeting any of the RIFLE criteria	Risk, 18.8 (6/32); injury, 15.6 (5/32); failure, 3.13 (1/32)
Akajagbor et al. (2013) (8)	67	1.5–2	Median, 123	Median, 74	Meeting any of the RIFLE criteria	Risk, 13.4 (9/67); injury, 19.4 (13/67); failure, 8.96 (6/67)
Phe et al. (2014) (10)	104		Mean, 104	Mean, 72	Meeting any of the RIFLE criteria	Risk, 4.8 (5/104); injury, 6.7 (7/104); failure, 11.5 (12/104)
Rigatto et al. (2016) (11)	410	1.5–3	Median, 150	Mean, 66	Meeting any of the RIFLE criteria	Risk, 22.4 (92/410); injury, 9 (45/410); failure, 12.7 (52/410)
Crass et al. (2017) (41)						
Non-cystic fibrosis patients	49	NA	Mean, 200.9	Mean, 83	Meeting any of the RIFLE criteria	Risk, 28.6 (14/49); injury, 12.2 (6/49); failure, 2.0 (1/49)
Cystic fibrosis patients	29	NA	Mean, 124.4	Mean, 55	Meeting any of the RIFLE Criteria	Risk, 24.1 (7/29); injury, 10.3 (3/29); failure, 0 (0/29)

a PMB, polymyxin B; CL_CR_, creatinine clearance (ml/min); SCr, serum creatinine (mg/dl); NA, not applicable; ↑, increase.

b Patients receiving polymyxin B who were also evaluated for nephrotoxicity.

**TABLE 2:** Summary of simulated ssAUC_0–24_ distributions for each study

Author (yr)	% of subjects with ≥25% decrease in CL_CR_	25th percentile ssAUC_0–24_ (mg · h/liter)	50th percentile ssAUC_0–24_ (mg · h/liter)	75th percentile ssAUC_0–24_ (mg · h/liter)
Ouderkirk et al. (2003)	NA^*a*^	43.1	57.4	73.9
Holloway et al. (2006)	22.5	47.7	67.4	90.6
Teng et al. (2007)	19.0	28.5	42.4	58.9
Pastewski et al. (2008)	18.0	48.1	48.1	60.1
Ramasubban et al. (2008)	9.0	46.6	59.7	81.0
Mendes et al. (2009)	22.0	37.5	50.8	70.2
Oliveira et al. (2009)	NA	35.8	51.0	69.2
Elias et al. (2010)	50.6	49.4	77.1	117
Kvitko et al. (2011)	35.0	51.1	75.7	108
Esaian et al. (2012)	48.0	46.8	54.2	67.6
Kubin et al. (2012)	60.0	45.3	62.2	88.0
Tuon et al. (2013)	20.8	52.5	76.8	105
Nandha et al. (2013)	19.0	39.3	57.0	79.8
Akajagbor et al. (2013)	41.8	47.6	62.3	76.4
Phe et al. (2014)	23.1	39.2	52.8	69.8
Rigatto et al. (2016)	46.1	64.0	83.4	111
Crass et al. (2017)				
Non-cystic fibrosis patients	42.9	60.6	81.0	110
Cystic fibrosis patients	34.5	61.4	79.8	101
Median (minimum–maximum)	28.8 (9.0–60.0)	47.2 (28.5–64.0)	61.0 (42.4–83.4)	80.4 (58.9–117)

a NA, not available.

**FIG 1:**
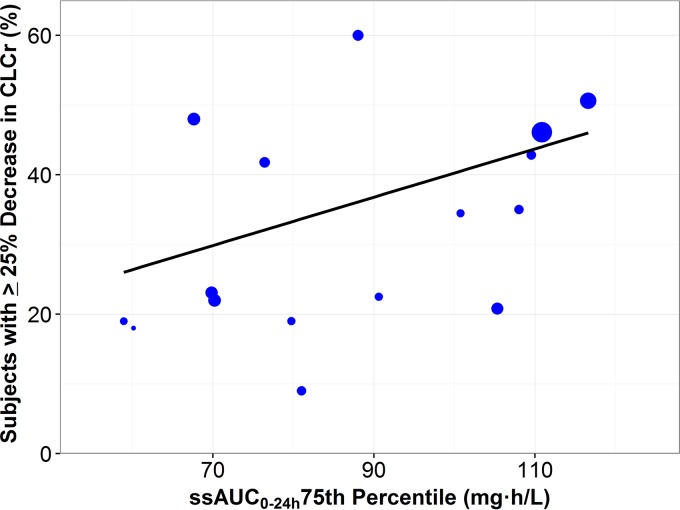
Portion of subjects with a ≥25% decrease in creatinine clearance plotted against the predicted polymyxin B ssAUC_0–24_ 75th percentile with an overlaid weighted linear regression. Each point represents a study; the size of the point is scaled according to the number of subjects in the study.

The portion of subjects with a ≥25% decrease in creatinine clearance plotted against the 75th percentile ssAUC_0–24_ with an overlaid weighted linear regression is shown in Fig. 1. Each point represents a study scaled according to study size. The linear regression (slope = 0.3465, intercept = 5.59, *P* = 0.0475) indicates a statistically significant (α < 0.05) relationship between nephrotoxicity and polymyxin B exposure. Overall, the studies by Elias et al. (19) and Rigatto et al. (11) had the highest simulated ssAUC_0–24_ (75th percentile = 117 and 111 mg · h/liter, respectively) and the second and fourth highest rates of nephrotoxicity, respectively. Similarly, the studies by Pastewski et al. (20) and Teng et al. (21) had the lowest simulated ssAUC_0–24_ (75th percentile = 60.1 and 58.9 mg · h/liter, respectively) and the second and third lowest rates of nephrotoxicity, respectively. These observations are consistent with the findings of the linear regression, which showed a significant relationship between nephrotoxicity and polymyxin B exposure. In the majority of these studies, the ssAUC_0–24_ values were below 100 mg · h/liter. The ssAUC_0–24_ that resulted in rates of mild nephrotoxicity (≤25% decreases in creatinine clearance [CL_CR_]) in ≤40% of subjects was determined to be 100 mg · h/liter. Given these findings, the upper edge of the polymyxin B target window, at this time, was estimated to be 100 mg · h/liter.

The lower bound of the therapeutic window was determined using data from previously described murine thigh infection model studies. In brief, Cheah et al. (15) evaluated colistin against Acinetobacter baumannii and Pseudomonas aeruginosa and identified free-drug plasma AUC_0–24_/MIC targets associated with 1-log_10_ CFU reductions from baseline that ranged from 3.5 to 13.9 and 6.6 to 10.9, respectively. More recently, Landersdorfer et al. (22) evaluated polymyxin B against Klebsiella pneumoniae and identified free-drug plasma AUC_0–24_/MIC targets associated with 1-log_10_ CFU reductions from baseline that ranged from 3.72 to 28.0. The median target across all three organisms evaluated in these two studies was a free-drug AUC_0–24_/MIC of 10.0. At the polymyxin breakpoint of 2 mg/liter for Enterobacteriaceae, P. aeruginosa, and A. baumannii, this median AUC/MIC target translates to a free-drug AUC_0–24_ target of 20 mg · h/liter. Using a 58% human protein binding for polymyxin B (23), this can be converted to a total-drug AUC_0–24_ of 47.6 mg · h/liter. Therefore, a total-drug plasma AUC of 47.6 mg · h/liter is associated with 1-log_10_ CFU reductions from baseline for isolates with MIC values of ≤2 mg/liter. We rounded this target to 50 mg · h/liter to simplify its use in a clinical setting.

In summary, given the results of the above-described toxicodynamic analysis and murine thigh infection models, the proposed target ssAUC_0–24_ window for polymyxin B is 50 to 100 mg · h/liter.

### Development and evaluation of adaptive feedback control algorithms.

The proposed adaptive feedback control algorithm was evaluated *in silico* using Monte Carlo simulations. In brief, a PK sample(s) collected with residual sampling error was simulated for each subject. The PK sample(s) along with the subject's weight were input into the MAP Bayesian estimator to compute an estimated clearance for each subject. A new, personalized dose was computed for each subject using the estimated clearance values and the midpoint of the target ssAUC_0–24_ window, 75 mg · h/liter. Subsequently, each subject's ssAUC_0–24_ was computed following administration of their personalized dosing regimen. Numerous PK sampling strategies, ranging from zero to four samples collected on day 1, were tested to evaluate improvements in target attainment relative to more intensive PK sampling schemes.

**FIG 2:**
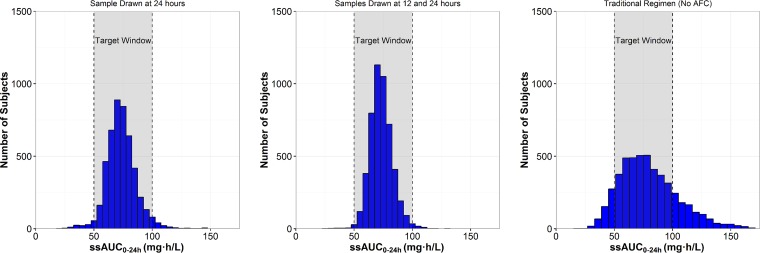
Histograms of ssAUC_0–24_ distributions relative to the target window following administration of new personalized polymyxin B doses computed using PK samples collected at different time points: a sample drawn at 24 h (left panel), samples drawn at 12 and 24 h (middle panel), and no samples (i.e., a traditional regimen, right panel). AFC, adaptive feedback control.

The results of these analyses are shown in Table 3. A histogram illustrating target attainment following administration of the new personalized doses is displayed in Fig. 2. Without adaptive feedback control (“one dose fits all”), only 71% of the 5,000 simulated subjects had ssAUC_0–24_ values within the target window of 50 to 100 mg · h/liter, and 19.8% of simulated subjects had ssAUC_0–24_ values above the target window. The total daily maintenance dose in the simulation utilizing zero PK samples was 2 mg/kg, which was computed using a target ssAUC_0–24_ of 75 mg · h/liter and a population average polymyxin B clearance of 0.0276 liters/h/kg. The maximum ssAUC_0–24_ obtained without adaptive feedback control was 223 mg · h/liter (over twice the upper bound of the target window). The percent coefficient of variation (%CV) for ssAUC_0–24_ without adaptive feedback control was 32.0%. Thus, “one dose fits all” provides no variability in dosing aside from accounting for patient weight and results in substantial imprecision in patient drug exposures. When personalized doses were computed using the adaptive feedback control algorithm with only one PK sample at either 12 or 24 h, 93% of simulated subjects achieved exposures within the target ssAUC_0–24_ window. As the number of PK samples increased, the probability of target attainment also increased. With four samples collected over the first 24 h of therapy, 99.3% of simulated subjects achieved an ssAUC_0–24_ within the target window (ssAUC_0–24_ %CV of 10.9%) following dose adjustments based on the adaptive feedback control algorithm. Personalized total daily doses of polymyxin B, after adaptive feedback control-based dose adjustment, varied nearly 14-fold (range, 0.50 to 6.8 mg/kg). The dose %CV was 29.7% with a single sample and 32.2% with four samples, indicating that a wide range of polymyxin B doses is required to achieve the target ssAUC_0–24_ across patients.

**TABLE 3:** Summary of simulations using 11 different sampling strategies utilizing 0 to 4 distinct PK samples with the adaptive feedback control algorithm

Sampling strategy	No. of PK samples	Sampling time(s) (h)	Probability of target window attainment (%)	% of subjects above target window	% of subjects below target window	Range of ssAUC_0–24_ values (mg · h/liter)	Range of adjusted polymyxin B doses (mg/kg/day)
1	0		71.0	19.8	9.2	19–223	2
2	1	12	93.6	5.0	1.4	17–153	0.77–4.6
3	1	24	95.3	2.5	2.2	22–195	0.83–4.4
4	2	2, 12	92.2	4.6	3.1	19–165	0.52–4.8
5	2	2, 24	96.5	1.6	1.9	19–140	0.66–4.9
6	2	4, 24	97.7	1.7	0.6	20–134	0.65–5.5
7	2	12, 24	98.5	0.9	0.6	27–128	0.67–5.3
8	3	2, 4, 12	93.7	4.1	2.2	21–155	0.50–5.2
9	3	2, 12, 24	99.2	0.5	0.3	21–118	0.68–5.0
10	3	4, 12, 24	99.5	0.4	0	23–119	0.64–6.8
11	4	2, 4, 12, 24	99.3	0.6	0	25–131	0.71–5.8

## DISCUSSION

Nephrotoxicity is the most clinically concerning polymyxin B toxicity, and it often limits the use of this therapy. It is unclear how best to optimize polymyxin B dosing regimens to achieve adequate drug exposure to treat life-threatening infections while avoiding nephrotoxic adverse events. Herein we investigated this problem *in silico* by first defining a polymyxin B target ssAUC_0–24_ window and then applying an adaptive feedback control algorithm to personalize dosing. In comparison to traditional polymyxin B dosing regimens, our strategy considerably increased the probability of achieving exposures within the target window.

Based on the pharmacometric meta-analysis of nephrotoxicity, the upper limit of the therapeutic window for polymyxin B was estimated to be an ssAUC_0–24_ of 100 mg · h/liter, which corresponds to an average steady-state polymyxin B plasma concentration of 4.2 mg/liter. This value was selected based on a linear function describing the relationship between nephrotoxicity rates and polymyxin B exposures. The question when selecting the upper bound of the therapeutic target window ultimately is what level of mild nephrotoxicity (i.e., ≥25% decrease in creatinine clearance) is acceptable, as we are unlikely to derive polymyxin B regimens which result in no risk of nephrotoxicity. An incidence of mild nephrotoxicity of less than 40% is a reasonable target, given that 63% of the studies evaluated in the meta-analysis described herein achieved this target. The lower bound of the target window was estimated to be an ssAUC_0–24_ of 50 mg · h/liter based on the results of published murine thigh infection model studies. The reader should also note that this target is adequate for the treatment of A. baumannii, K. pneumoniae, and P. aeruginosa isolates with MIC values of ≤2 mg/liter. This is reasonable, given the MIC_90_ values for A. baumannii and P. aeruginosa (2 and 2 mg/liter, respectively) (24) and the current published breakpoints for these organisms (2 mg/liter) (25). This is the first publication to define a therapeutic target window for performing polymyxin B dose optimization using an adaptive feedback control algorithm. However, it is important to recognize some of the limitations of our current study. Given that individual patient data regarding polymyxin B dose optimization and associated changes in renal function were not available in the literature, our analysis relied on the summary statistics reported for these measures in each of the studies included in our meta-analysis. Ultimately, this resulted in a less robust data set than would have been ideal. Additionally, considerable between-subject variability was observed between the studies evaluated due, in part, to factors such as various levels of concomitantly administering nephrotoxic drugs. This led to significant variability in the nephrotoxicity rates that could not be explained by polymyxin B exposure alone and which likely confounded the results of our analyses. As more clinical data become available, the upper bound of the polymyxin B therapeutic window should be reevaluated.

Using the derived polymyxin B target ssAUC_0–24_ window, an adaptive feedback control algorithm was evaluated using Monte Carlo simulation with various sparse PK sampling strategies. Sampling windows were constrained to the first day of treatment on the basis of the facts that (i) polymyxin B rapidly decreases bacterial density (13), (ii) nephrotoxicity often manifests within the first several days of therapy (26), and (iii) early optimization of antibiotic regimens is critical (27). By collecting samples on day 1, all necessary adjustments to the dosing regimen can be performed within 1 to 2 days of the start of therapy depending on the availability, location, and turnaround times of polymyxin B assays. Therefore, by day 2 or 3 of therapy, patients could receive a personalized dosing regimen. We also evaluated sampling times between 2 and 12 h to determine if dose optimization could occur even earlier.

Our simulations indicated that without adaptive feedback control, only 71% of subjects achieved ssAUC_0–24_ values within the target therapeutic window of 50 to 100 mg · h/liter (corresponding to average steady-state concentrations of 2.1 to 4.2 mg/liter). With a single 24-h PK sample and adaptive feedback control-based dose adjustments, the probability of target attainment increased to over 95%. Slightly fewer subjects (93.6%) attained the target when the single PK sample was drawn at 12 h rather than at 24 h. However, the overall clinical and economic benefits of optimizing doses 12 h earlier may outweigh this slight deficit in the number of subjects achieving the targeted exposures. Most importantly, subjects with dose adjustments who did not attain the target window still had ssAUC_0–24_ values that were close to the target ssAUC_0–24_ window (Fig. 2), whereas subjects without adaptive feedback control exhibited a much wider ssAUC_0–24_ distribution (%CV, 17.4% versus 32.0%, respectively). Additionally, a substantial number of subjects (*n* = 73) had AUC values above 150 mg · h/liter, an exposure 50% greater than the upper bound of the target window, when adaptive feedback control was not utilized. With personalized dosing using adaptive feedback control, patients are much more likely to be treated effectively with reduced risk of nephrotoxicity even if they do not achieve the target ssAUC_0–24_ window.

Polymyxin B dose optimization can be performed with only a single PK sample obtained on the first day of therapy. Additional clinical trials are warranted to demonstrate that the target ssAUC_0–24_ can be achieved by adaptive feedback control utilizing limited PK sampling schemes and to provide evidence that the approach described herein is able to improve clinical outcomes. Given the vulnerability of patients receiving polymyxin B, the potential consequences of under- or overdosing patients, and the low number of PK samples needed, individualized polymyxin B dosing using the proposed adaptive feedback control algorithm is a clinically viable and appealing solution that could help improve clinical practice.

## MATERIALS AND METHODS

### Pharmacometric meta-analysis of nephrotoxicity.

The PubMed database was searched in December 2017 to identify any study published in English reporting intravenous polymyxin B nephrotoxicity rates. “Polymyxin,” “polymyxin B,” “nephrotoxicity,” “adverse event,” and “toxicity” were used as search terms. The following information was gathered from each publication when available: definition of nephrotoxicity, number of subjects and nephrotoxicity events, dosing guidelines used, and descriptive statistics about the polymyxin doses administered and body weight. Studies were excluded if details provided regarding the polymyxin B dosing regimen reported were inadequate or if the nephrotoxicity rate associated with intravenous polymyxin B was unclear. Using the data gathered from the included studies, a pharmacometric meta-analysis was performed to produce a toxicodynamic model to relate polymyxin B exposure to nephrotoxicity (28).

The included studies used inconsistent criteria to describe nephrotoxicity events. Many studies simply looked at the incidence of any nephrotoxic event, while others reported the degree or grade of nephrotoxicity. The RIFLE (risk, injury, failure, loss of kidney function, and end-stage kidney disease) criteria were commonly used. RIFLE defines renal risk, injury, and failure as a 25% decrease in creatinine clearance or a 1.5- to 2-fold increase in serum creatinine, a 50% decrease in creatinine clearance or a 2- to 3-fold increase in serum creatinine, and a 75% decrease in creatinine clearance or >3-fold increase in serum creatinine, respectively (29). The RIFLE criteria were used as a common metric to unify the collected nephrotoxicity data. In our analysis, three grades of nephrotoxicity, ≥25%, ≥50%, and ≥75% decreases in creatinine clearance (CL_CR_) from baseline, were used. Each group represented the cumulative number of subjects meeting the classification criteria.

Duration of treatment, AUC, and baseline CL_CR_ drive polymyxin nephrotoxicity (15–17). Similarly, polymyxin B nephrotoxicity is related to both the daily dose and the duration of treatment (26). Given this, AUC is likely associated with polymyxin B nephrotoxicity. Using data from the collected studies, the relationship between observed nephrotoxicity rates and the predicted polymyxin B AUC was explored. To perform this analysis, expected polymyxin B AUC values were derived for each study using Monte Carlo simulations. Specifically, the simulations were performed using the polymyxin B population PK model developed by Sandri et al. (23) and the patient descriptors and polymyxin B dosing information collected from each study. For each study, the 25th, 50th, and 75th percentiles of the simulated steady-state AUC values from 0 to 24 h (ssAUC_0–24_) and the cumulative proportion of subjects in each nephrotoxicity category were computed. Relationships between the selected ssAUC_0–24_ percentiles and each nephrotoxicity category were examined using unweighted and weighted linear and nonlinear regressions. The results of the toxicodynamic analysis were used to propose the upper bound of the target ssAUC_0–24_ window for polymyxin B.

### Development and evaluation of adaptive feedback control algorithms.

A linear, two-compartment population PK model previously developed by Sandri et al. (23), using PK samples (eight samples per subject) collected from 24 critically ill patients, was used as the adaptive feedback control algorithm backbone. Total body weight was a significant covariate on all parameters. The population mean clearance was 0.0276 liters/h/kg with between-subject variability of 32.4%. To test the adaptive feedback control algorithm, Monte Carlo simulations of proposed PK sampling strategies, with various numbers of samples ranging from zero (no optimization) to four, were implemented. Each simulation consisted of 5,000 subjects receiving a traditional polymyxin B regimen of a 2.5 mg/kg loading dose infused over 2 h, followed by 1.5 mg/kg infused over 1 h twice daily. All plasma concentrations were simulated with random measurement error. For each sampling strategy, subject-specific polymyxin B plasma concentrations were provided as input to the MAP Bayesian estimator. Subject-specific clearance values were estimated and used to compute new, personalized polymyxin B doses. The new personalized daily doses were computed by dividing the target ssAUC_0–24_ (middle of target window) by the subject-specific estimated polymyxin B clearance. A final ssAUC_0–24_ (after the dose adjustment) was estimated for each subject by dividing the new personalized polymyxin B dose by the subject's true clearance. The percentage of subjects with ssAUC_0–24_ values within the proposed target window, also known as the probability of target attainment, was determined for the various sampling strategies, including the traditional dosing regimen with no PK sampling.

Modeling and simulations were implemented in ADAPT 5 (30). All graphical and statistical analyses were performed in R version 3.2.1 for Windows (31).
